# METABOLOMICS BIOMARKERS FOR FRAILTY: A LONGITUDINAL STUDY OF AGING MICE

**DOI:** 10.1093/geroni/igae098.2768

**Published:** 2024-12-31

**Authors:** Dantong Zhu, Judy Wu, Brady Samuelson, Alice Kane

**Affiliations:** Institute for Systems Biology, Seattle, Washington, United States; Institute for Systems Biology, Seattle, Washington, United States; Institute for Systems Biology, Seattle, Washington, United States; Institute for Systems Biology, Seattle, Washington, United States

## Abstract

Aging is associated with changes in dynamic biological processes which may contribute to the development of aging-related diseases. Metabolomic profiling allows us to measure a wide spectrum of metabolites as read outs of these biological processes. The temporal dynamics of metabolites can be utilized to identify age-related biomarkers and to elucidate possible aging mechanisms. We performed a longitudinal study of aging female (n = 20) and male (n = 25) C57 mice and measured frailty index and derived metabolomics from plasma samples at five time points from 13 to 30 months of age. We identify differentially abundant metabolites by differential analysis over the time course and select metabolite features by a machine learning approach both in the whole cohort and in sex-stratified subgroups. We reveal novel metabolites associated with △frailty index (difference between individual frailty index and median frailty index at a given sex and age group), and merge these metabolites with frailty index and age related metabolites to generate a union set of metabolite features associated with frailty in aging. We found frailty related metabolites are generally enriched in lipid metabolism, amino acid metabolism and metabolism of cofactors and vitamins, although there is strong sex dimorphism of the specific metabolites. Notably, we identify FAD and 4−hydroxyhippurate in females and ergothioneine and creatine in males as candidate metabolites associated with frailty. In summary, our results identify sex-specific metabolite biomarkers of frailty in aging, and shed light on potential mechanisms involved in aging and the development of frailty.

